# Supplementary material for: Extracellular fibrinogen-binding protein released by intracellular Staphylococcus aureus suppresses host immunity by targeting TRAF3
Source: Nat Commun. 2022 Sep 19;13:5493. doi: 10.1038/s41467-022-33205-z (PMC9484707; doi:10.1038/s41467-022-33205-z)
Supplement: Supplementary file 1 — Supplementary Information [file 41467_2022_33205_MOESM1_ESM.pdf]

Supplementary Information for

Extracellular fibrinogen-binding protein released by intracellular *Staphylococcus aureus* suppresses host immunity by targeting TRAF3

Xiaokai Zhang[1,6], Tingrong Xiong[1,6], Lin Gao[1,6], Yu Wang[1,2,6], Luxuan Liu[3], Tian Tian[1], Yun Shi[4], Jinyong Zhang[1], Zhuo Zhao[1], Dongshui Lu[1], Ping Luo[1], Weijun Zhang[1], Ping Cheng[1], Haiming Jing[1], Qiang Gou[1], Hao Zeng[1]*, Dapeng Yan[5]*, Quanming Zou[1]*.

[1]National Engineering Research Center of Immunological Products, Department of Microbiology and Biochemical Pharmacy, College of Pharmacy, Third Military Medical University, Chongqing 400038, China.
[2]Department of Basic Courses, NCO School, Third Military Medical University, Shijiazhuang 050081, China.
[3]College of Medicine, Southwest Jiaotong University, Chengdu 610083, China.
[4]Institute of Biopharmaceutical Research, West China Hospital, Sichuan University, Chengdu, Sichuan 610041, China.
[5]Department of Immunology, School of Basic Medical Sciences, Shanghai Institute of Infectious Disease and Biosecurity & Shanghai Public Health Clinical Center, Fudan University, Shanghai 200032, China
[6] These authors contributed equally: Xiaokai Zhang, Tingrong Xiong, Lin Gao, Yu Wang.
*These authors jointly supervised this work: Hao Zeng, Dapeng Yan, Quanming Zou; e-mail: qmzou2007@163.com; dapengyan@fudan.edu.cn; zeng1109@163.com.

This PDF file includes:

Supplementary Fig. 1 to 16.

Supplementary Table 1-2.

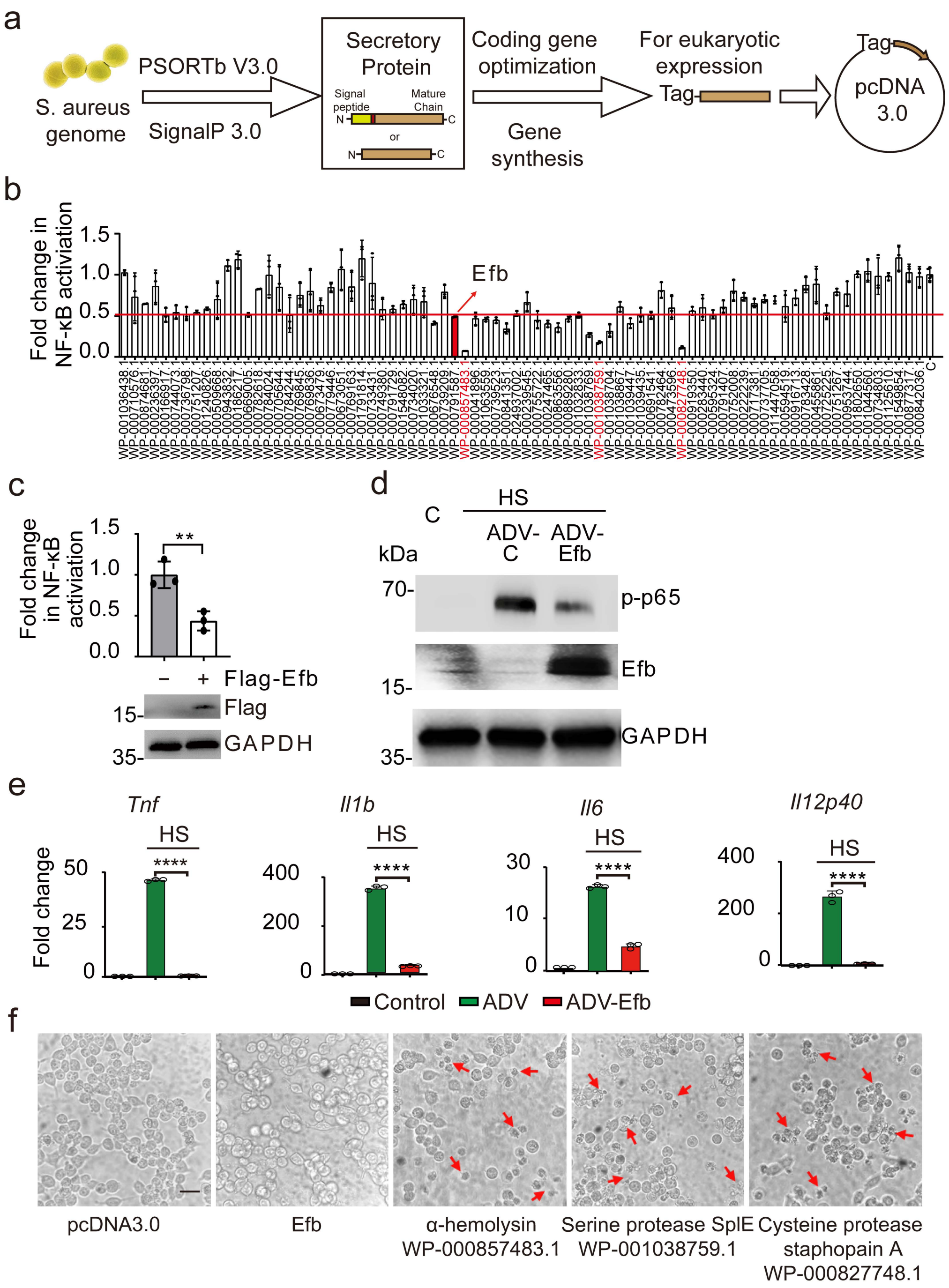

Supplementary Fig. 1. Efb inhibits NF-κB activation. a, Vector construction methods. **b**, Luciferase assay of HEK 293T cells transfected using NF-κB luciferase reporters with 78 *S. aureus* secreted effector-encoding plasmids for 24 h (***P*=0.0084). **c**, Luciferase assay and related immunoblot of HEK293T cells transfected using NF-κB luciferase reporters encoding plasmids with or without Efb-encoding plasmids

for 24 h. **d**, Immunoblot of WCL of PMs transduced using control adenovirus (ADV) or Efb-encoding ADV for 48 h, and then stimulated by heat killed *S. aureus* (HS, MOI=25) for 6 h. **e**, qPCR analysis of *Tnf*, *Il1b*, *Il6*, and *Il12p40* mRNA from PMs transduced with control ADV or Efb-encoding ADV for 48 h and then stimulated with heat killed *S. aureus* (HS, MOI=25) for 6 h (****P<0.0001, *Tnf*; ****P<0.0001, *Il1b*; ****P<0.0001, *Il6*; ****P<0.0001, *Il12p40*). **f**, Cell morphology of HEK 293T cells (scale bars, 25 μm). Student's two-tailed unpaired t-test (b, c, e) was used for statistical analysis. Data are representative of three experiments with at least three independent biological replicates. The bars show the mean and standard deviation of n=3 (b, c, e). Source data are provided as a Source Data file.

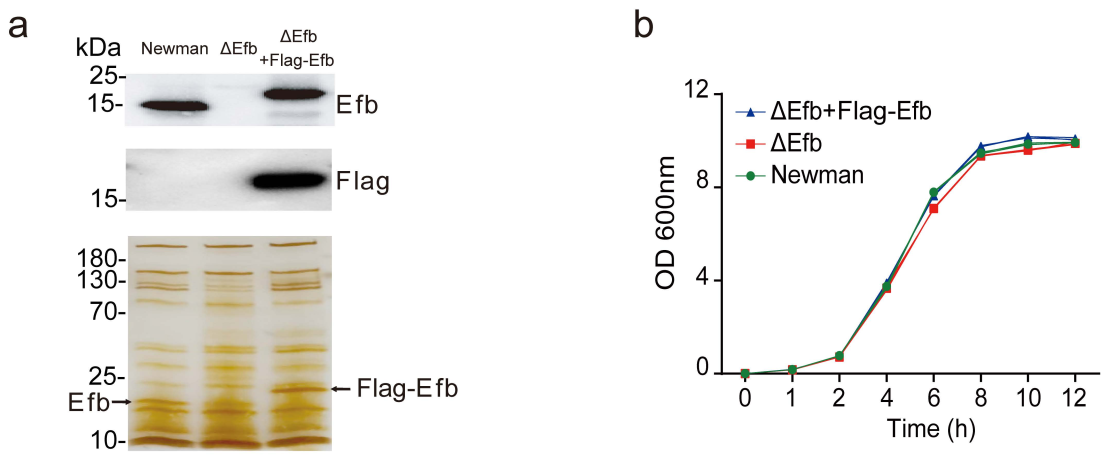

Supplementary Fig. 2. Validation of Efb mutant strains. a, Immunoblot and silver staining of culture supernatant of Newman, ΔEfb, and ΔEfb+Flag-Efb for 12 h. **b**, Growth curve of Newman, ΔEfb, and Δ Efb+Flag-Efb. Data are representative of three experiments with at least three independent biological replicates. Source data are provided as a Source Data file.

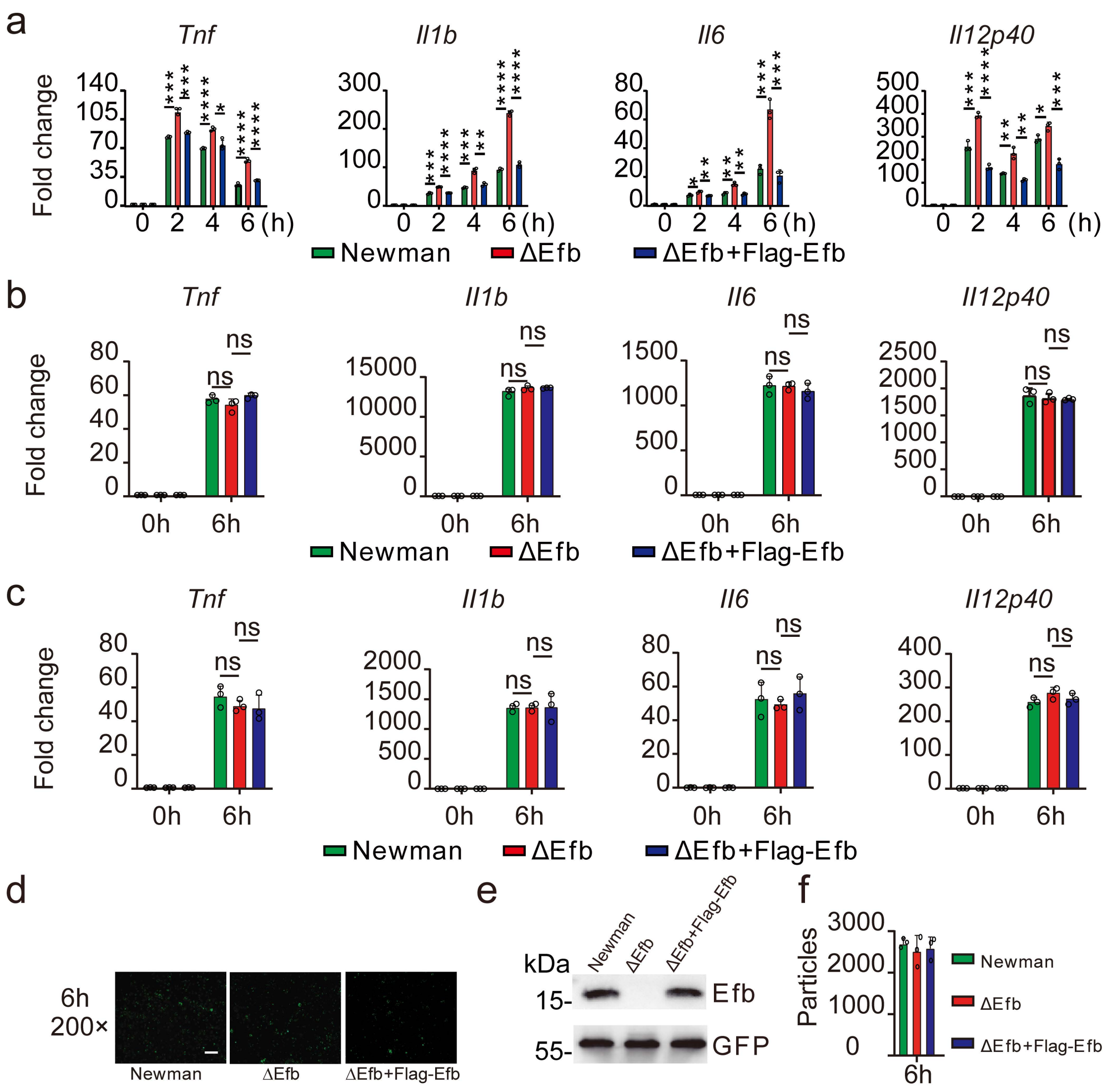

Supplementary Fig. 3. Efb inhibits host pro-inflammatory responses. a, qPCR analysis of *Tnf*, *Il1b*, *Il6*, and *Il12p40* mRNA from PMs infected by Newman, ΔEfb, or ΔEfb+Flag-Efb for indicated times (MOI=25; ****P*=0.0001, 0.0005, *****P*<0.0001, **P*=0.0117, *****P*<0.0001, <0.0001 in sequence, *Tnf*; ****P*=0.0003, *****P*<0.0001, ****P*=0.0004, ***P*=0.0014, *****P*<0.0001, <0.0001, in sequence, *Il1b*; **P*=0.0154, ***P*=0.0026, 0.0020, 0.0012, ****P*=0.0007, 0.0006, in sequence, *Il6*; ****P*=0.0008,

****$P<0.0001$, **$P=0.0037$, 0.0014, *$P=0.0140$, ***$P=0.0006$, in sequence, *Il12p40*). **b,** qPCR analysis of *Tnf*, *Il1b*, *Il6*, and *Il12p40* mRNA from PMs stimulated by culture supernatant of Newman, ΔEfb, or ΔEfb+Flag-Efb. **c,** qPCR analysis of *Tnf*, *Il1b*, *Il6*, and *Il12p40* mRNA from PMs infected by Newman, ΔEfb, or ΔEfb+Flag-Efb for indicated times using non-contact co-culture methods (Transwell 0.4 μm, MOI=25). **d,** Intracellular survival of Newman, ΔEfb, or ΔEfb+Flag-Efb (scale bars, 100 μm). **e**, Immunoblots of culture supernatants of Newman, ΔEfb, or ΔEfb+Flag-Efb expressing GFP for 12 h. **f,** Intracellular survival of Newman, ΔEfb, or ΔEfb+Flag-Efb (MOI=25). Student's two-tailed unpaired t-test (a, b, c, f) was used for statistical analysis. Data are representative of three experiments with at least three independent biological replicates. The bars show the mean and standard deviation of n=3 (a, b, c, f). Source data are provided as a Source Data file.

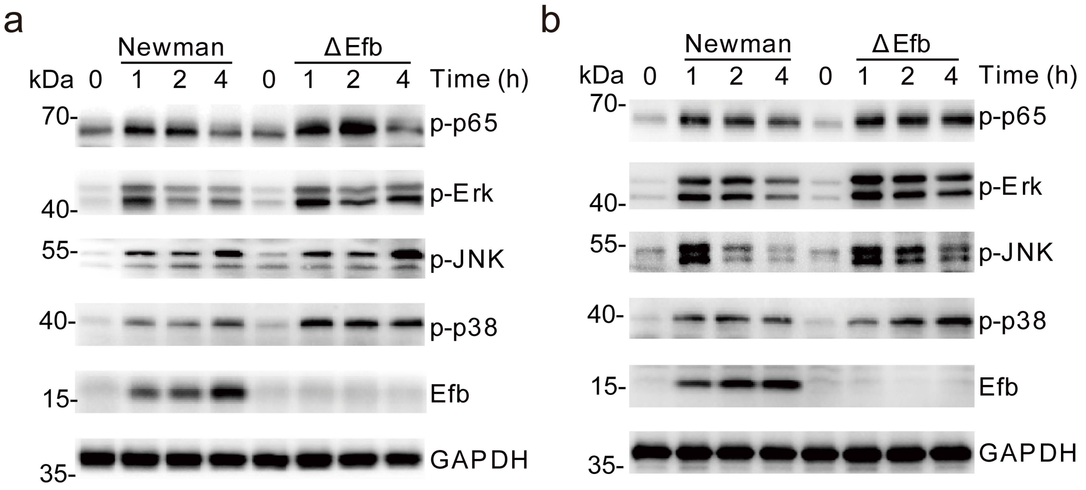

Supplementary Fig. 4. Efb inhibits pro-inflammatory signaling pathways of macrophages. a, b, Immunoblot of MH-S, PMs infected by Newman or ΔEfb for indicated times (MOI=25). Data are representative of three experiments with at least three independent biological replicates. Source data are provided as a Source Data file.

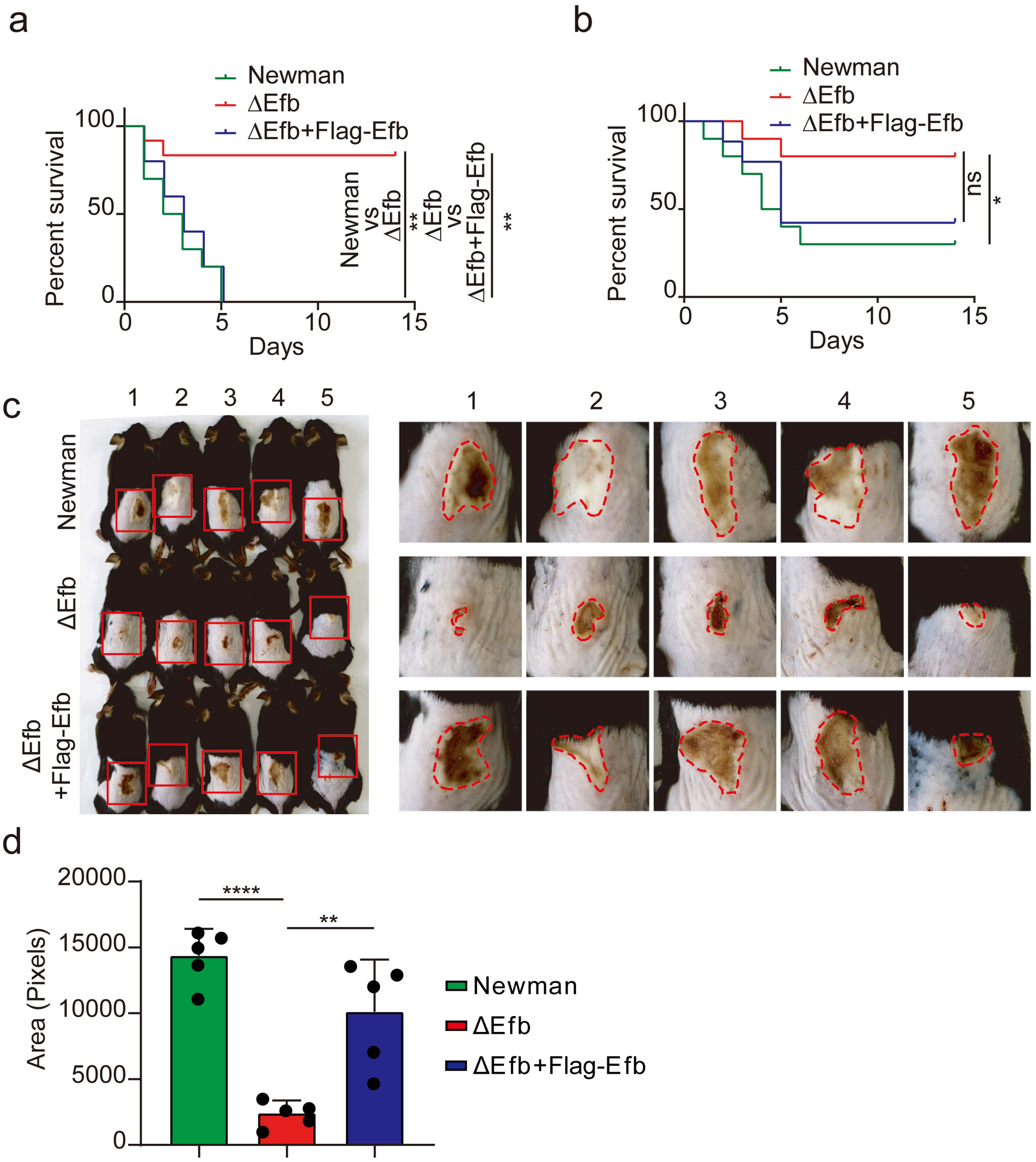

Supplementary Fig. 5. Efb is an important effector for *S. aureus* infection. a, Survival rates of 6-week-old mice infected by intratracheal administration with roughly 6×10^{8} CFUs per mouse of Newman, ΔEfb, or ΔEfb+Flag-Efb (***P*=0.0046, Newman vs ΔEfb; ***P*=0.0069, ΔEfb vs ΔEfb+Flag-Efb). **b**, Survival of 6-week-old mice infected by tail vein injection with roughly 2×10^{8} CFUs per mouse of Newman, ΔEfb, or ΔEfb+Flag-Efb (**P*=0.0254, Newman vs ΔEfb; *P*=0.0873, ΔEfb vs

ΔEfb+Flag-Efb). **c, d,** Skin lesion size of 6-week-old mice infected by subcutaneous injection with roughly 1×10^8 CFUs per mouse of Newman, ΔEfb, or ΔEfb+Flag-Efb (*****P*<0.0001,***P*=0.0029). Two-tailed Gehan-Breslow-Wilcoxon test (a, b, n=8) and Student's two-tailed unpaired t-test (d, n=5) were used for statistical analysis. Data are representative of three experiments with at least three independent biological replicates. The bars show the mean and standard deviation of n=3 (d). Source data are provided as a Source Data file.

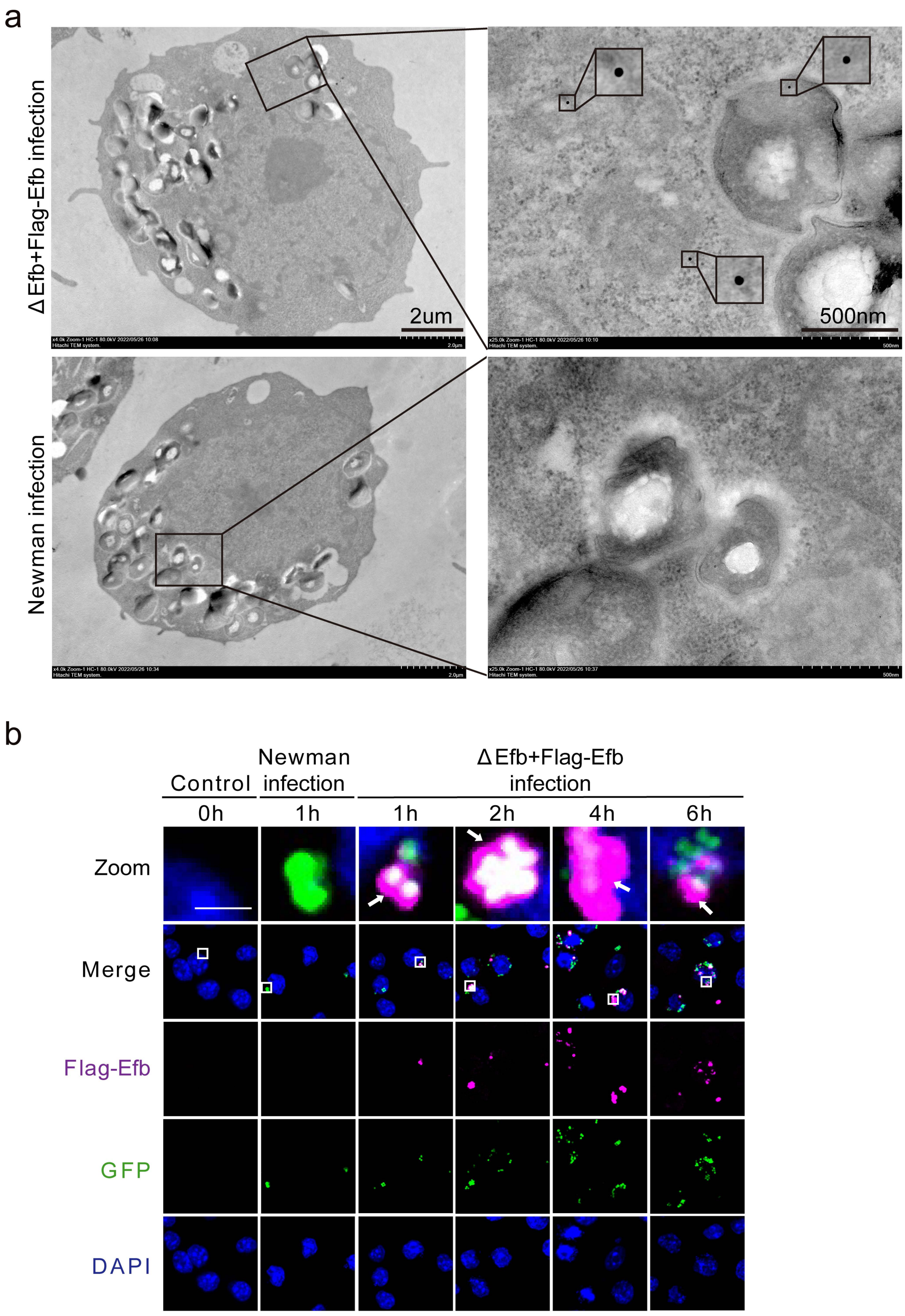

Supplementary Fig. 6. Intracellular *S. aureus* secretes Efb into macrophage cytoplasm. a, Immunoelectronmicroscopy assay of MH-S infected with Newman and ΔEfb + Flag-Efb at 4 h. **b**, Immunofluorescence assay of MH-S infected with Newman and ΔEfb + Flag-Efb expressed GFP for indicated times (MOI=25), scale

bars, 2 μm. Data are representative of three experiments with at least three independent biological replicates.

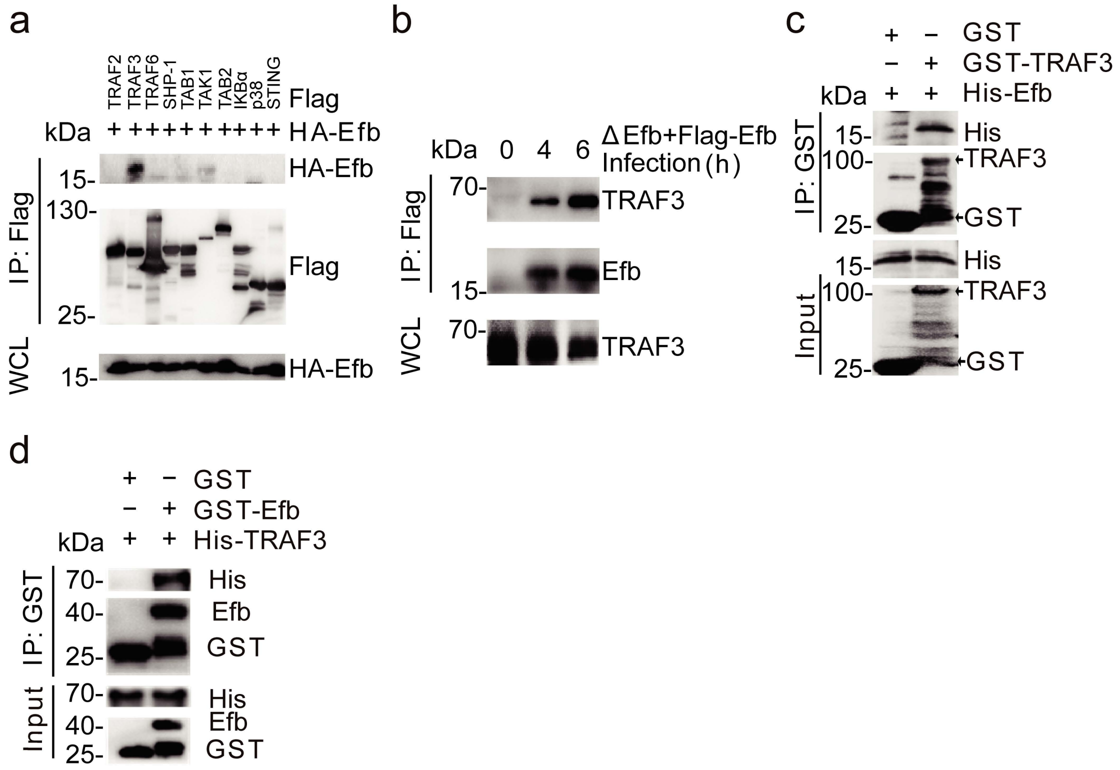

Supplementary Fig. 7. Efb interacts with TRAF3.a, Immunoblots of WCL and IP products from HEK 293T cells transfected with indicated plasmids. **b**, Immunoblots of WCL and IP products from PMs infected with ΔEfb + Flag-Efb for indicated times (MOI=25). **c**, **d**, Precipitation assay of purified TRAF3 and purified Efb. Data are representative of three experiments with at least three independent biological replicates. Source data are provided as a Source Data file.

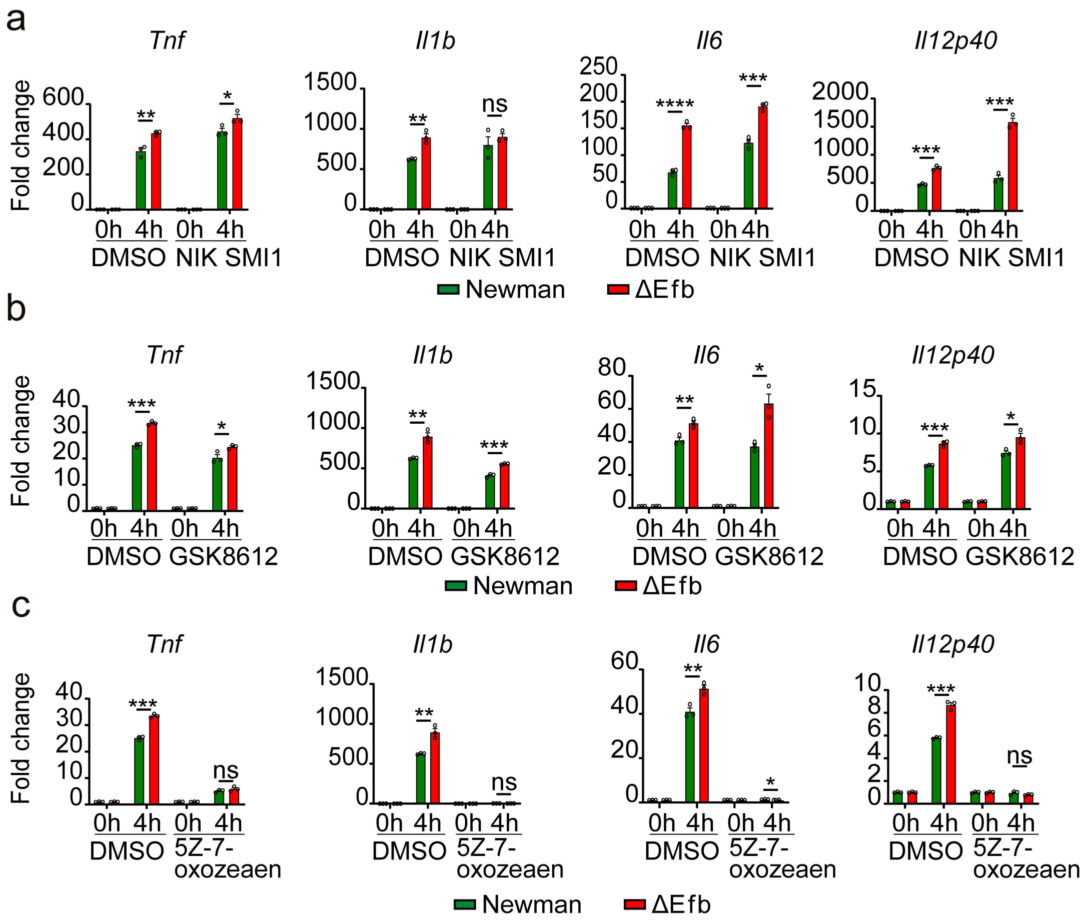

Supplementary Fig. 8. Efb inhibits host pro-inflammatory responses mainly through canonical NF-kB and MAPK pathways. a, b, c, qPCR analysis of *Tnf*, *Il1b*, *Il6*, and *Il12p40* mRNA from MH-S infected by Newman or ΔEfb for indicated times (MOI=25), (**P=0.0068, *P=0.0470, in sequence, *Tnf*; **P=0.0040, *Il1b*; ****P<0.0001, ***P=0.0007, in sequence, *Il6*; ***P=0.0003, 0.0003, in sequence, *Il12p40*; in **a**), (***P=0.0002, *P=0.0352, in sequence, *Tnf*; **P=0.0040, ***P=0.0003, in sequence, *Il1b*; **P=0.0096, *P=0.0108, in sequence, *Il6*; ***P=0.0002, *P=0.0216, in sequence, *Il12p40*; in **b**), (***P=0.0002, *Tnf*; **P=0.0040, *Il1b*; **P=0.0096, *P=0.0477, in sequence, *Il6*; ***P=0.0002, *Il12p40*; in **c**). Student's two-tailed unpaired t-test (a, b, c) was used for statistical analysis. Data are representative of three experiments with at least three independent biological replicates. The bars show the mean and standard deviation of n=3 (a, b, c). Source data are provided as a Source Data file.

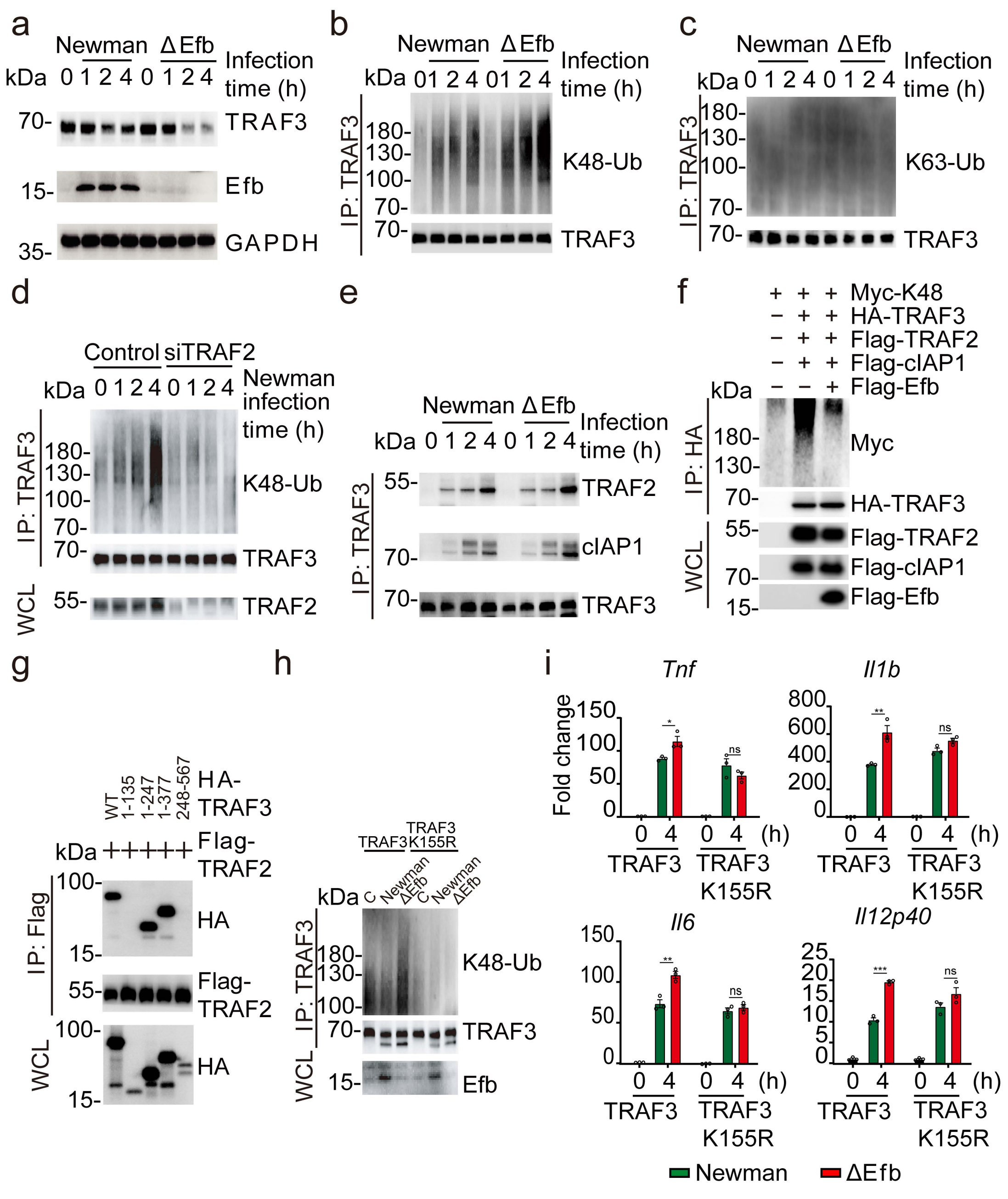

Supplementary Fig. 9. Efb stabilizes TRAF3. **a**, Immunoblots of lysates from PMs infected with Newman and ΔEfb for indicated times (MOI=25). **b**, **c**, Immunoblots of IP products of WCL from PMs infected with Newman and ΔEfb for indicated times (MOI=25). **d**, Immunoblots of WCL and IP products from lysates of MH-S infected

with Newman and ΔEfb for indicated times (MOI=25). **e**, Immunoblots of IP products from PMs infected with Newman and ΔEfb for indicated times. **f**, **g,** Immunoblots of WCL and IP products from HEK 293T cells transfected with indicated plasmids. **h**, Immunoblots of WCL and IP products from lysates of TRAF3 KO PMs (transfected with TRAF3 or TRAF3K155R mRNA) infected with Newman and ΔEfb for indicated times (MOI = 25). **i**, qPCR analysis of *Tnf*, *Il1b*, *Il6*, and *Il12p40* mRNA from TRAF3 KO PMs infected by Newman, ΔEfb for indicated times (MOI=25; **P*=0.0302, *Tnf*; ***P*=0.0084, *Il1b*; ***P*=0.0057, *Il6*; ****P*=0.0001, *Il12p40*). Student's two-tailed unpaired t-test (**i**) was used for statistical analysis. Data are representative of three experiments with at least three independent biological replicates. The bars show the mean and standard deviation of n=3 (**i**). Source data are provided as a Source Data file.

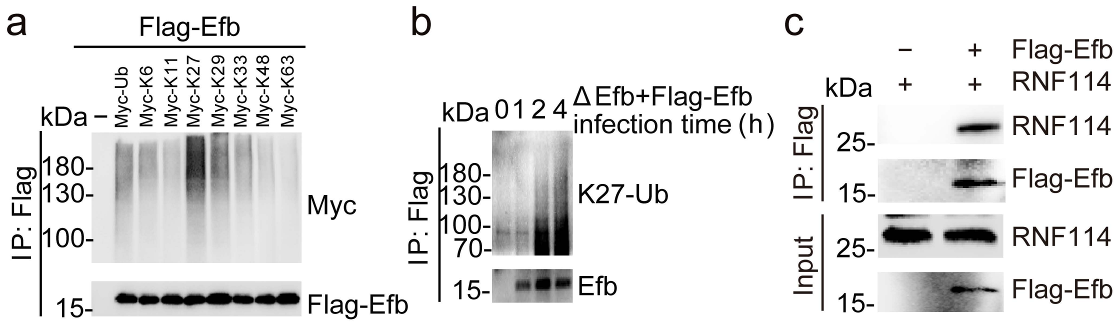

Supplementary Fig. 10. Efb is K27-ubiquitinated by host RNF114. **a**, Immunoblots of IP products from lysates of HEK 293T cells transfected with indicated plasmids. **b**, Immunoblots of IP products from WCL of PMs infected with ΔEfb+Flag-Efb for indicated times (MOI=25). **c**, Immunoblots of IP products culture supernatant ΔEfb+Flag-Efb strain and WCL of MH-S. Data are representative of three experiments with at least three independent biological replicates. Source data are provided as a Source Data file.

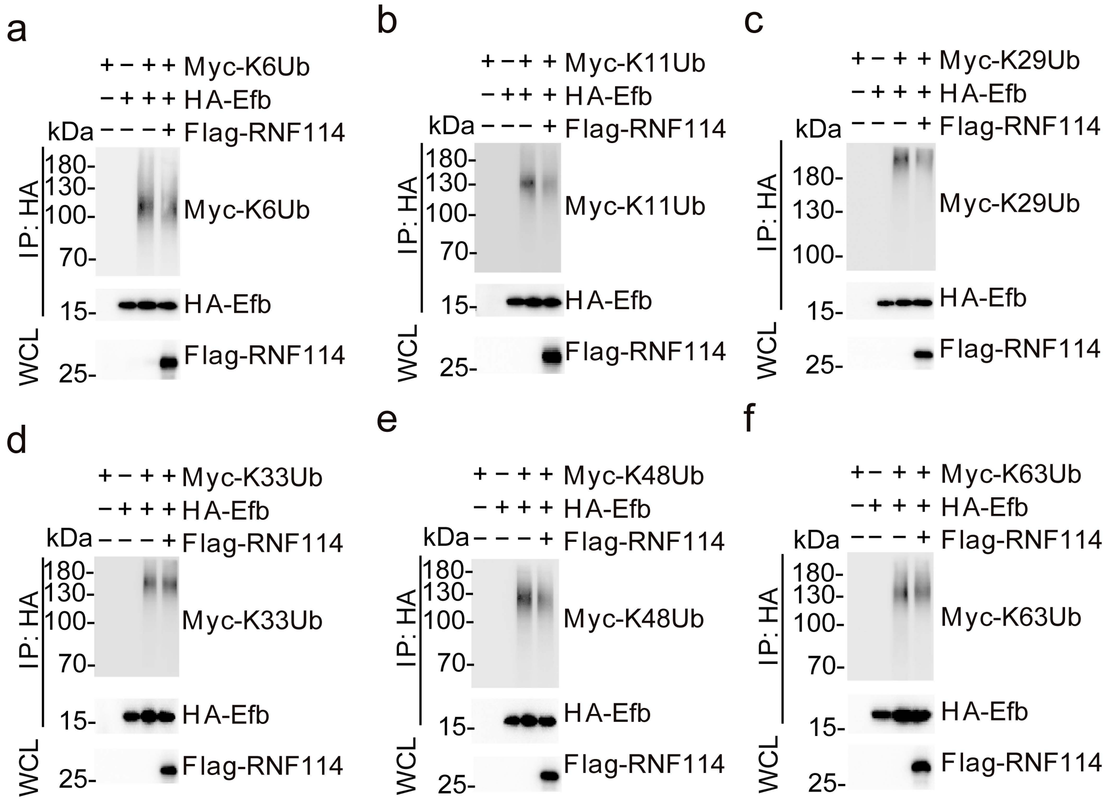

Supplementary Fig. 11. RNF114 cannot promote K6, K11, K29, K33, K48, and K63 linked polyubiquitination of Efb. a, **b**, **c**, **d**, **e**, **f**, Immunoblots of WCL and IP products of WCL of HEK 293T cells transfected with indicated plasmids. Data are representative of three experiments with at least three independent biological replicates. Source data are provided as a Source Data file.

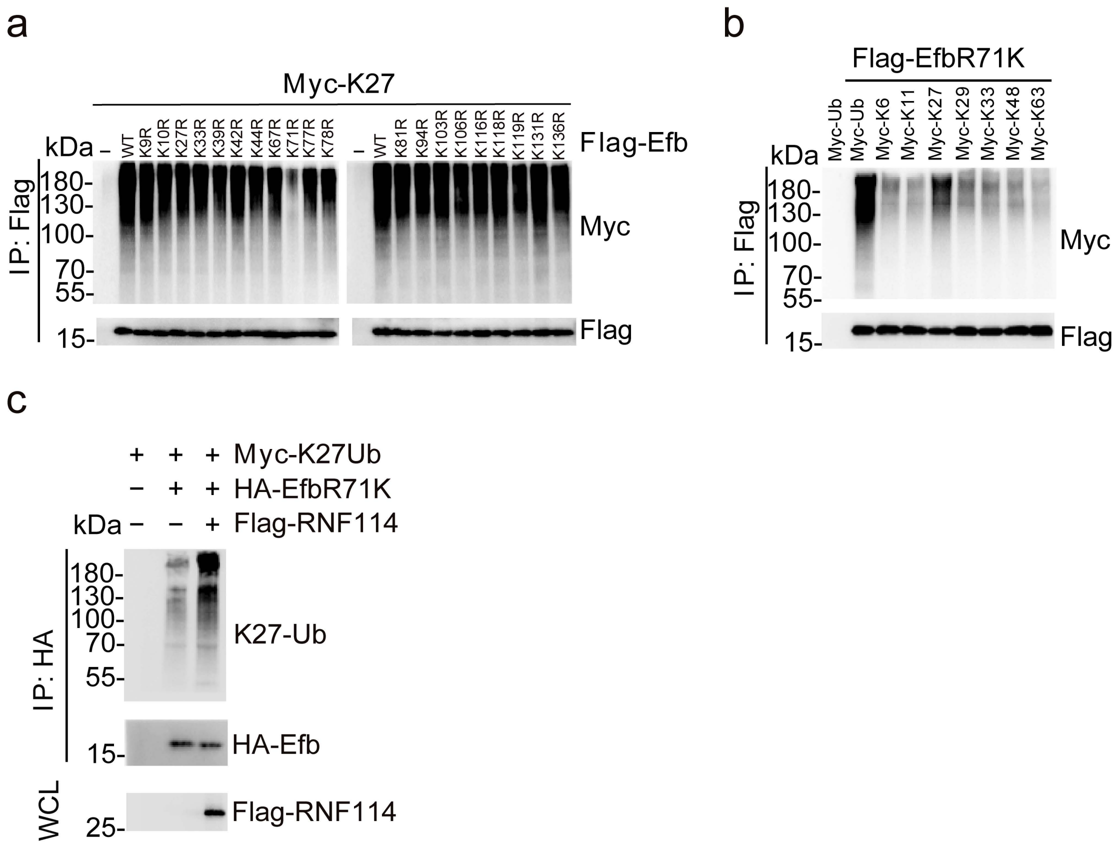

Supplementary Fig. 12. RNF114 promotes K27-ubiquitination of Efb at K71. a, **b**, **c**, Immunoblots of WCL and IP products of WCL of HEK 293T cells transfected with indicated plasmids. K71R, replace 71 lysine of Efb with arginine. R71K, replace 71 arginine of mutant of Efb (replace all lysine with arginine) with lysine. Data are representative of three experiments with at least three independent biological replicates. Source data are provided as a Source Data file.

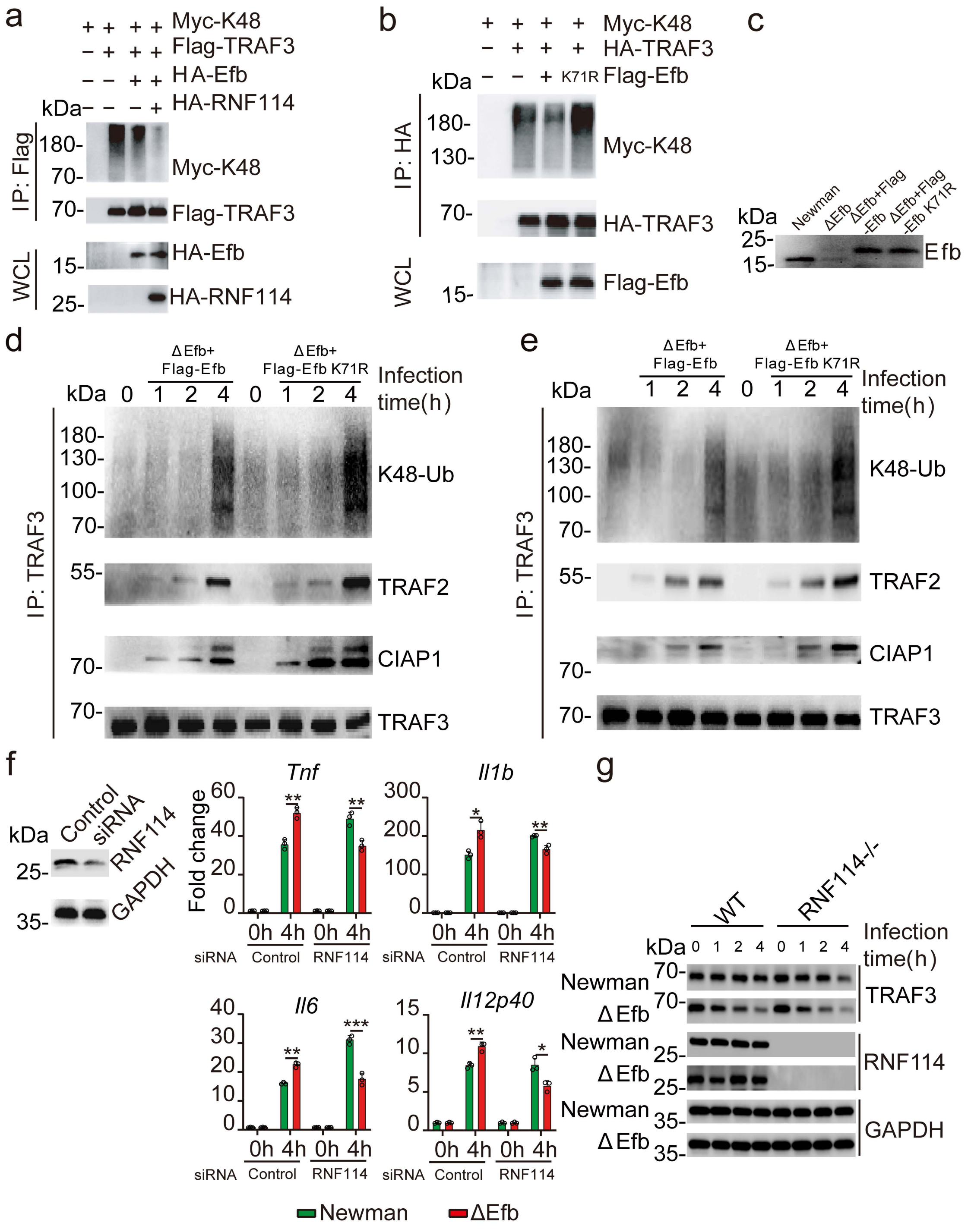

Supplementary Fig. 13. Ubiquitinated Efb facilitates TRAF3 stabilization. a, b,

Immunoblots of WCL and IP products of WCL of HEK 293T cells transfected with indicated plasmids. K71R, replace 71 lysine of Efb with arginine. **c**, Immunoblots of culture supernatant of *S. aureus* for 12 h. **d**, **e**, Immunoblots of IP products of WCL from PMs or MH-S infected with ΔEfb + Flag-Efb or ΔEfb + Flag-Efb K71R for indicated times. **f**, qPCR analysis of *Tnf*, *Il1b*, *Il6*, and *Il12p40* mRNA from control or RNF114 KD MH-S infected by Newman and ΔEfb for indicated times (MOI=25; ***P*=0.0017, ***P*=0.0051, in sequence, *Tnf*; **P*=0.0103, ***P*=0.0059, in sequence, *Il1b*; ****P*=0.0003, 0.0007, in sequence, *Il6*; ***P*=0.0030, **P*=0.0136, in sequence, *Il12p40*). **g**, Immunoblots of WCL from PMs infected with Neman and ΔEfb for indicated times. Student's two-tailed unpaired t-test (f) was used for statistical analysis. Data are representative of three experiments with at least three independent biological replicates. The bars show the mean and standard deviation of n=3 (f). Source data are provided as a Source Data file.

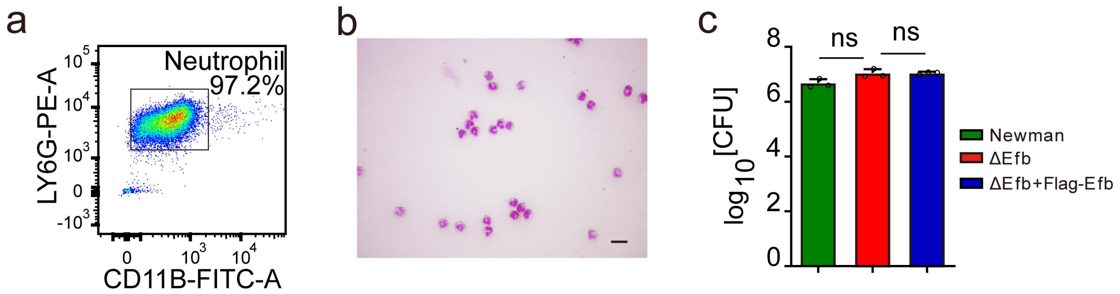

Supplementary Fig. 14. Neutrophil killing assay. **a**, Flow cytometry analysis of isolated cells. **b**, Wright's staining of isolated cells (scale bar, 25 μm). **c**, Survival of Newman, ΔEfb, or ΔEfb+Flag-Efb cultured with neutrophils (MOI=25). Two tailed Mann-Whitney U test (c) was used for statistical analysis. Data are representative of three experiments with at least three independent biological replicates. The bars show the mean and standard deviation of n=3. Source data are provided as a Source Data file.

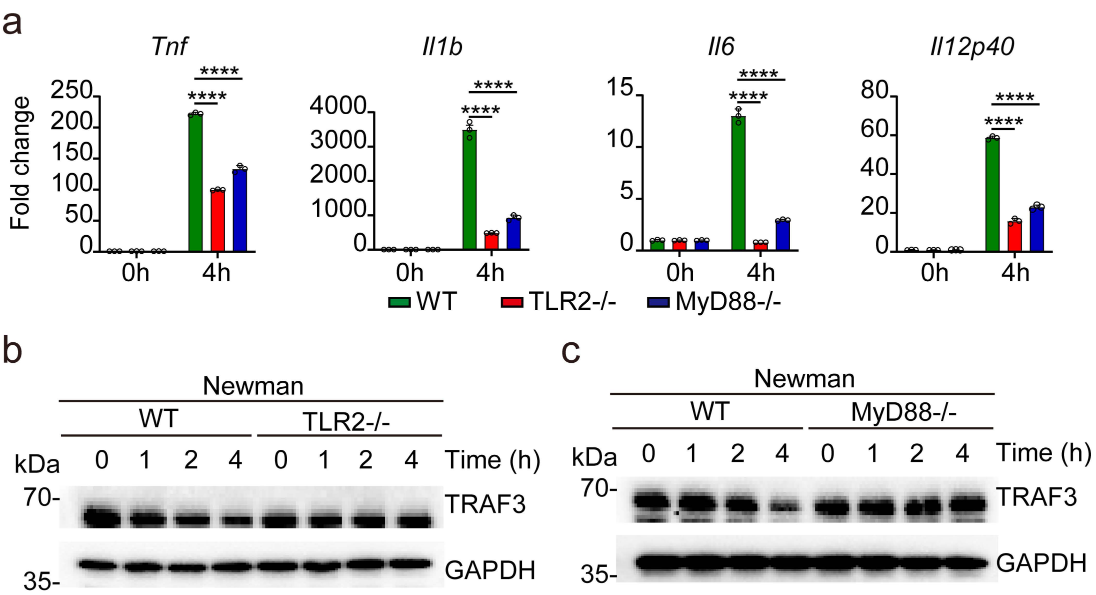

Supplementary Fig. 15. TLR2 and MyD88 mediate macrophage host pro-inflammatory responses caused by *S. aureus*. a, qPCR analysis of *Tnf*, *Il1b*, *Il6*, and *Il12p40* mRNA from PMs infected with Newman for indicated times (MOI=25; *****P*<0.0001, *Tnf*; *****P*<0.0001, *Il1b*; *****P*<0.0001, *Il6*; *****P*<0.0001, *Il12p40*). **b**, Immunoblots of WCL from PMs infected with Neman and ΔEfb for indicated times. Student's two-tailed unpaired t-test (a) was used for statistical analysis. Data are representative of three experiments with at least three independent biological replicates. The bars show the mean and standard deviation of n=3. Source data are provided as a Source Data file.

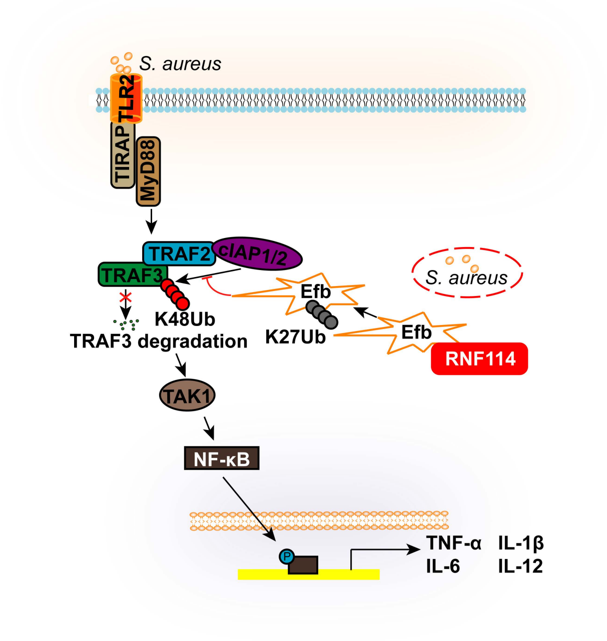

Supplementary Fig. 16. RNF114 mediated K27-linked ubiquitination (K27Ub) of Efb suppresses the TLR2/TRAF3-mediated expression of pro-inflammatory cytokines (TNF-α, IL-1β, IL-6, and IL-12) by disturbing formation of the TRAF3/TRAF2/cIAP1 complex.

Supplementary table1. Bacterial strains and plasmids used in this study

Name (Strains/Plasmids/Primers)	Description	Reference
E. coli DH5α	F-φ80lacZ ΔM15 (lacZYA-argF) U169 endA1 recA1hsdR17 (rk-,mk+) supE44λ-thi-1 gyrA96 relA1 phoA	Tiangen
E. coli BL21	F-ompT hsdSB (rB- mB-) gal dcm (DE3)	Tiangen
S. aureus	RN4200	X. Rao
S. aureus	Newman	X. Rao
Newman GFP	Integration of sarA_P1-CFP-Term downstream gene NWMN_0029	This study
Newman ΔEfb	Newman strain with deletion of Efb	This study
Newman ΔEfb GFP	Integration of sarA_P1-CFP-Term downstream gene NWMN_0029	This study
ΔEfb+Flag-Efb	Newman ΔEfb strain complemented with wild type Efb (Flag tag on N terminal)	This study
ΔEfb+Flag-Efb GFP	Integration of sarA_P1-CFP-Term downstream gene NWMN_0029	This study
ΔEfb+Flag-Efb K71R	Newman ΔEfb strain complemented with Efb K71R (Flag tag on N terminal)	This study
pBT2	*S. aureus*-*E. coli* shuttle vector, temperature sensitive, AmpR in E. coli and CmR in S. aureus	X. Rao
pLI50	S. aureus-E. coli shuttle overexpressed vector, AmpR in E. coli and CmR in S. aureus	X. Rao
pLI50-Flag-Efb	For expression of Efb fused to an N-terminal Flag tag in Newman ΔEfb	This study
pLI50-Flag-Efb K71R	For expression of Efb K71R fused to an N-terminal Flag tag in Newman ΔEfb	This study
pTH100-GFP	pJB38-NWMN29-30 + SarA_P1-sGFP-Term	Addgene plasmid #84458
pET28a-Efb	For expression of *S. aureus* Efb protein fused to an N-terminal his6 tag in BL21	This study
pET28a-TRAF3	For expression of mouse TRAF3 protein fused to an N-terminal his6 tag in BL21	This study
pGEX-6p-1-Efb	For expression of *S. aureus* Efb protein fused to an N-terminal GST tag in BL21	This study
pGEX-6p-1-TRAF3	For expression of mouse TRAF3 protein fused to an N-terminal GST tag in BL21	This study
pcDNA3.0-Flag-TRAF3	For expression of mouse TRAF3 protein fused to an N-terminal GST tag in HEK 293T cells	This study
pcDNA3.0-HA-TRAF3	For expression of mouse TRAF3 protein fused to an N-terminal HA tag in HEK 293T cells	This study
pcDNA3.0-HA-TRAF3 (1-135)	For expression of mouse TRAF3 protein (1-135) fused to an N-terminal HA tag in HEK 293T cells	This study
pcDNA3.0-HA-TRAF3 (1-247)	For expression of mouse TRAF3 protein (1-247) fused to an N-terminal HA tag in HEK 293T cells	This study
pcDNA3.0-HA-TRAF3 (1-377)	For expression of mouse TRAF3 protein (1-377) fused to an N-terminal HA tag in HEK 293T cells	This study
pcDNA3.0-HA-TRAF3 (248-567)	For expression of mouse TRAF3 protein (248-567) fused to an N-terminal HA tag in HEK 293T cells	This study
pcDNA3.0-Flag-TRAF3 (K/R)	For expression of mouse TRAF3 protein (all lysine replaced with arginine) fused to an N-terminal Flag tag in HEK 293T cells	This study
pcDNA3.0-Flag-TRAF3 (R106K)	For expression of mouse TRAF3 (K/R) (106 lysine replaced with arginine) fused to an N-terminal Flag tag in HEK 293T cells	This study
pcDNA3.0-Flag-TRAF3 (R155K)	For expression of mouse TRAF3 (K/R) (155 lysine replaced with arginine) fused to an N-terminal Flag tag in HEK 293T cells	This study
pcDNA3.0-Flag-TRAF3 (R106/155K)	For expression of mouse TRAF3 (K/R) (106 and 155 lysine replaced with arginine) fused to an N-terminal Flag tag in HEK 293T cells	This study
pcDNA3.0-Flag-Efb	For expression of *S. aureus* Efb fused to an N-terminal Flag tag in HEK 293T cells	This study
pcDNA3.0-HA-Efb	For expression of *S. aureus* Efb fused to an N-terminal HA tag in HEK 293T cells	This study
pcDNA3.0-Flag-Efb K9R	For expression of *S. aureus* Efb (9 lysine replaced with arginine) fused to an N-terminal Flag tag in HEK 293T cells	This study
pcDNA3.0-Flag-Efb K10R	For expression of *S. aureus* Efb (10 lysine replaced with arginine) fused to an N-terminal Flag tag in HEK 293T cells	This study
pcDNA3.0-Flag-Efb K27R	For expression of *S. aureus* Efb (27 lysine replaced with arginine) fused to an N-terminal Flag tag in HEK 293T cells	This study
pcDNA3.0-Flag-Efb K33R	For expression of *S. aureus* Efb (33 lysine replaced with arginine) fused to an N-terminal Flag tag in HEK 293T cells	This study
pcDNA3.0-Flag-Efb K39R	For expression of *S. aureus* Efb (39 lysine replaced with arginine) fused to an N-terminal Flag tag in HEK 293T cells	This study
pcDNA3.0-Flag-Efb K42R	For expression of *S. aureus* Efb (42 lysine replaced with arginine) fused to an N-terminal Flag tag in HEK 293T cells	This study
pcDNA3.0-Flag-Efb K44R	For expression of *S. aureus* Efb (44 lysine replaced with arginine) fused to an N-terminal Flag tag in HEK 293T cells	This study
pcDNA3.0-Flag-Efb K67R	For expression of *S. aureus* Efb (67 lysine replaced with arginine) fused to an N-terminal Flag tag in HEK 293T cells	This study
pcDNA3.0-Flag-Efb K71R	For expression of *S. aureus* Efb (71 lysine replaced with arginine) fused to an N-terminal Flag tag in HEK 293T cells	This study
pcDNA3.0-Flag-Efb K77R	For expression of *S. aureus* Efb (77 lysine replaced with arginine) fused to an N-terminal Flag tag in HEK 293T cells	This study
pcDNA3.0-Flag-Efb K78R	For expression of *S. aureus* Efb (78 lysine replaced with arginine) fused to an N-terminal Flag tag in HEK 293T cells	This study
pcDNA3.0-Flag-Efb K81R	For expression of *S. aureus* Efb (81 lysine replaced with arginine) fused to an N-terminal Flag tag in HEK 293T cells	This study
pcDNA3.0-Flag-Efb K94R	For expression of *S. aureus* Efb (94 lysine replaced with arginine) fused to an N-terminal Flag tag in HEK 293T cells	This study
pcDNA3.0-Flag-Efb K103R	For expression of *S. aureus* Efb (103 lysine replaced with arginine) fused to an N-terminal Flag tag in HEK 293T cells	This study
pcDNA3.0-Flag-Efb K106R	For expression of *S. aureus* Efb (106 lysine replaced with arginine) fused to an N-terminal Flag tag in HEK 293T cells	This study
pcDNA3.0-Flag-Efb K116R	For expression of *S. aureus* Efb (116 lysine replaced with arginine) fused to an N-terminal Flag tag in HEK 293T cells	This study
pcDNA3.0-Flag-Efb K118R	For expression of *S. aureus* Efb (118 lysine replaced with arginine) fused to an N-terminal Flag tag in HEK 293T cells	This study
pcDNA3.0-Flag-Efb K119R	For expression of *S. aureus* Efb (119 lysine replaced with arginine) fused to an N-terminal Flag tag in HEK 293T cells	This study
pcDNA3.0-Flag-Efb K131R	For expression of *S. aureus* Efb (131 lysine replaced with arginine) fused to an N-terminal Flag tag in HEK 293T cells	This study
pcDNA3.0-Flag-Efb K136R	For expression of *S. aureus* Efb (136 lysine replaced with arginine) fused to an N-terminal Flag tag in HEK 293T cells	This study

pcDNA3.0-HA-Efb K/R	For expression of *S. aureus* Efb (all lysine replaced with arginine) fused to an N-terminal HA tag in HEK 293T cells	This study
pcDNA3.0-HA-Efb R71K	For expression of *S. aureus* Efb K/R (71 arginine replaced with lysine) fused to an N-terminal HA tag in HEK 293T cells	This study
pcDNA3.0-Flag-RNF114	For expression of mouse RNF114 fused to an N-terminal Flag tag in HEK 293T cells	This study
pcDNA3.0-HA-RNF114	For expression of mouse RNF114 fused to an N-terminal HA tag in HEK 293T cells	This study
pcDNA3.0-Flag-TRAF2	For expression of mouse TRAF2 fused to an N-terminal Flag tag in HEK 293T cells	This study
pcDNA3.0-Flag-TRAF6	For expression of mouse TRAF6 fused to an N-terminal Flag tag in HEK 293T cells	This study
pcDNA3.0-Flag-SHP-1	For expression of mouse SHP-1 fused to an N-terminal Flag tag in HEK 293T cells	This study
pcDNA3.0-Flag-TAB1	For expression of mouse TAB1 fused to an N-terminal Flag tag in HEK 293T cells	This study
pcDNA3.0-Flag-TAK1	For expression of mouse TAK1 fused to an N-terminal Flag tag in HEK 293T cells	This study
pcDNA3.0-Flag-IκBα	For expression of mouse IκBα fused to an N-terminal Flag tag in HEK 293T cells	This study
pcDNA3.0-Flag-p38	For expression of mouse p38 fused to an N-terminal Flag tag in HEK 293T cells	This study
pcDNA3.0-Flag-STING	For expression of mouse Sting fused to an N-terminal Flag tag in HEK 293T cells	This study
pcDNA3.0-Myc-Ub	For expression of mouse Ub fused to an N-terminal Myc tag in HEK 293T cells	This study
pcDNA3.0-Myc-Ub (K6)	For expression of mouse Ub (K6) fused to an N-terminal Myc tag in HEK 293T cells	This study
pcDNA3.0-Myc-Ub (K11)	For expression of mouse Ub (K11) fused to an N-terminal Myc tag in HEK 293T cells	This study
pcDNA3.0-Myc-Ub (K27)	For expression of mouse Ub (K27) fused to an N-terminal Myc tag in HEK 293T cells	This study
pcDNA3.0-Myc-Ub (K29)	For expression of mouse Ub (K29) fused to an N-terminal Myc tag in HEK 293T cells	This study
pcDNA3.0-Myc-Ub (K33)	For expression of mouse Ub (K33) fused to an N-terminal Myc tag in HEK 293T cells	This study
pcDNA3.0-Myc-Ub (K48)	For expression of mouse Ub (K48) fused to an N-terminal Myc tag in HEK 293T cells	This study
pcDNA3.0-Myc-Ub (K63)	For expression of mouse Ub (K63) fused to an N-terminal Myc tag in HEK 293T cells	This study
pcDNA3.0-HA-Ub	For expression of mouse Ub fused to an N-terminal HA tag in HEK 293T cells	This study
pcDNA3.0-HA-Ub (K48)	For expression of mouse Ub (K48) fused to an N-terminal HA tag in HEK 293T cells	This study
pcDNA3.0-HA-Ub (K63)	For expression of mouse Ub (K63) fused to an N-terminal HA tag in HEK 293T cells	This study
pNF-κB–luc	For screening effectors of *S. aureus* on activation or inhibition NF-κB pathway	D. Yan
pRL–TK	A control for pNF-κB–luc	D. Yan

Supplementary table2. Primers used in this study

mTnfF	TTCTGTCTACTGAACTTCGGGGTGATCGGTCC
mTnfR	GTATGAGATAGCAAATCGGCTGACGGTGTGGG
mIL6F	CTGCAAGAGACTTCCATCCAG
mIL6R	AGTGGTATAGACAGGTCTGTTGG
mGAPDHF	TGGCCTTCCGTGTTCCTAC
mGAPDHR	GAGTTGCTGTTGAAGTCGCA
mIL12bF	GTCCTCAGAAGCTAACCATCTCC
mIL12bR	CCAGAGCCTATGACTCCATGTC
mIL1bF	GAAATGCCACCTTTTGACAGTG
mIL1bR	TGGATGCTCTCATCAGGACAG
ΔEfb-F1	CATCGCAGTGCAGCGGAATTCTGGTTGATATGTTATGAT
ΔEfb-R1	CAGGTCGACTCTAGAGGATCCGTTAATTATCCTCCAAATTAT
ΔEfb-F2	ATTTGGAGGATAATTAACGGATCCAACTTCAATCGTTGCTGTTATC
ΔEfb-R2	CTTGCATGCCTGCAGGTCGACGTGGCCCCGCCAACT
