# Supplementary material for: Extracellular fibrinogen-binding protein released by intracellular Staphylococcus aureus suppresses host immunity by targeting TRAF3
Source: Nat Commun. 2022 Sep 19;13:5493. doi: 10.1038/s41467-022-33205-z (PMC9484707; doi:10.1038/s41467-022-33205-z)
Supplement: Supplementary file 5 — Reporting Summary [file 41467_2022_33205_MOESM5_ESM.pdf]

nature portfolio

Corresponding author(s): Hao Zeng, Dapeng Yan, Quanming Zou

Last updated by author(s): Aug 28, 2022

Reporting Summary

Nature Portfolio wishes to improve the reproducibility of the work that we publish. This form provides structure for consistency and transparency in reporting. For further information on Nature Portfolio policies, see our Editorial Policies and the Editorial Policy Checklist.

Statistics

For all statistical analyses, confirm that the following items are present in the figure legend, table legend, main text, or Methods section.

n/a	Confirmed	
☐	☒	The exact sample size (n) for each experimental group/condition, given as a discrete number and unit of measurement
☐	☒	A statement on whether measurements were taken from distinct samples or whether the same sample was measured repeatedly
☐	☒	The statistical test(s) used AND whether they are one- or two-sided<br>*Only common tests should be described solely by name; describe more complex techniques in the Methods section.*
☒	☐	A description of all covariates tested
☒	☐	A description of any assumptions or corrections, such as tests of normality and adjustment for multiple comparisons
☐	☒	A full description of the statistical parameters including central tendency (e.g. means) or other basic estimates (e.g. regression coefficient) AND variation (e.g. standard deviation) or associated estimates of uncertainty (e.g. confidence intervals)
☐	☒	For null hypothesis testing, the test statistic (e.g. F, t, r) with confidence intervals, effect sizes, degrees of freedom and P value noted<br>*Give P values as exact values whenever suitable.*
☒	☐	For Bayesian analysis, information on the choice of priors and Markov chain Monte Carlo settings
☒	☐	For hierarchical and complex designs, identification of the appropriate level for tests and full reporting of outcomes
☒	☐	Estimates of effect sizes (e.g. Cohen's d, Pearson's r), indicating how they were calculated

Our web collection on statistics for biologists contains articles on many of the points above.

Software and code

Policy information about availability of computer code

Data collection	The Immunohistochemistry data:ZEN 2.1 on Zeiss LSM 780<br>The qRT-PCR data: Bio-Rad CFX Manager 3.1 on Bio-Rad CFX96<br>The western blot data: Image Lab TM Touch Software 1.2 on ChemiDoc TM Touch Imaging System
Data analysis	GraphPad Prism 8.0<br>Image J 1.8 for calculating fluorescent dots<br>Photoshop cc2018 for calculating skin area

For manuscripts utilizing custom algorithms or software that are central to the research but not yet described in published literature, software must be made available to editors and reviewers. We strongly encourage code deposition in a community repository (e.g. GitHub). See the Nature Portfolio guidelines for submitting code & software for further information.

Data

Policy information about availability of data

All manuscripts must include a data availability statement. This statement should provide the following information, where applicable:

- Accession codes, unique identifiers, or web links for publicly available datasets
- A description of any restrictions on data availability
- For clinical datasets or third party data, please ensure that the statement adheres to our policy

All data are available within the present article and supplementary Information or Source Data files.

Field-specific reporting

Please select the one below that is the best fit for your research. If you are not sure, read the appropriate sections before making your selection.

☒ Life sciences ☐ Behavioural & social sciences ☐ Ecological, evolutionary & environmental sciences

For a reference copy of the document with all sections, see nature.com/documents/nr-reporting-summary-flat.pdf

Life sciences study design

All studies must disclose on these points even when the disclosure is negative.

Sample size	For animal assay, sample sizes were selected empirically from previous experimental experience with similar assays, and/or from sizes generally employed in the field. For cell assay,sizes were selected empirically from previous experimental experience with similar assays.
Data exclusions	No exclusion of data points or images were used.
Replication	Data are representative of at least 3 independent experiments, and all attempts at replication were successful.
Randomization	For mice infection experiments, six-week-old C57BL/6 female mice were divided randomly into cages and infected with different S. aureus strains. S. aureus were allocated into groups according genotype of interest.
Blinding	Investigators were blinded during data collection and analysis where possible.

Reporting for specific materials, systems and methods

We require information from authors about some types of materials, experimental systems and methods used in many studies. Here, indicate whether each material, system or method listed is relevant to your study. If you are not sure if a list item applies to your research, read the appropriate section before selecting a response.

Materials & experimental systems

n/a	Involved in the study	
☐	☒	Antibodies
☐	☒	Eukaryotic cell lines
☒	☐	Palaeontology and archaeology
☐	☒	Animals and other organisms
☒	☐	Human research participants
☒	☐	Clinical data
☒	☐	Dual use research of concern

Methods

n/a	Involved in the study	
☒	☐	ChIP-seq
☐	☒	Flow cytometry
☒	☐	MRI-based neuroimaging

Antibodies

Antibodies used	Rabbit anti-TRAF3 (PA5-20165, Invitrogen; ab36988, Abcam), mouse anti-TRAF3 (sc6933, Santa Cruz), rabbit anti-TRAF2 (4724, Cell signaling technology, CST), mouse anti-Flag (F1804, Sigma-Aldrich), rabbit anti-HA (3724, CST), rabbit anti-Myc (2040, CST), rabbit anti-phospho-p65 (3033, CST), rabbit anti-phospho-p38 (9215, CST), rabbit anti-phospho-Erk1/2 (9101, CST), rabbit anti-phospho-JNK (4668, CST), rabbit anti-GFP (2956, CST), rabbit anti-K27 (ab181537, Abcam), rabbit anti-K48 (8081, CST), rabbit anti-K63 (5621), rabbit anti-cIAP1 (ab2399, Abcam), rabbit anti-RNF114 (ab97303, Abcam), Alexa Fluor Plus 488 conjugated goat anti-mouse IgG (A32723, Invitrogen), Alexa Fluor Plus 555 conjugated goat anti-mouse IgG (A32727, Invitrogen), Alexa Fluor Plus 647 conjugated goat anti-rabbit IgG (A32733, Invitrogen); Anti-Efb was generated by immunization of rabbits with the protein of Efb, rabbit anti-GST (CW0085M, Cwbio), rabbit anti-His (CW0083M Cwbio); rabbit anti-GAPDH (G9545, Sigma-Aldrich), mouse anti-Flag M2 Affinity Gel (A2220, Sigma-Aldrich), mouse anti-HA Magnetic Beads (88836, Thermo Fisher), mouse anti-TRAF3 agarose beads (sc-6933 AC, Santa Cruz), and goat anti-rabbit IgG (5127, CST), goat anti-mouse IgG (96714, CST). rat anti-mouse Ly-6G PE (551461, BD), rat anti-mouse CD11b-FITC (557396, BD).
Validation	The commercial antibodies are well used and reported in lots of previous publications. Rabbit anti-TRAF3 (PA5-20165)) is used for human and mouse species for Western blot, Immunocytochemistry, Immunofluorescence; mouse anti-TRAF3 (sc6933) is used for mouse, rat and human species for Western blot, Immunoprecipitation, Immunofluorescence, Flow cytometry; rabbit anti-TRAF2 (4724) is used for mouse, huamn and monkey species for Western blot, Immunoprecipitation, Immunofluorescence, Flow cytometry; mouse anti-Flag (F1804) is used for detection of Flag peptide in Western blot, Immunoprecipitation, Immunofluorescence; rabbit anti-HA (3724) is used for detection of HA peptide in Western blot, Immunoprecipitation, Immunofluorescence, Flow cytometry; rabbit anti-Myc (2040) is used for detection of Myc peptide in Western blot, Immunoprecipitation, Immunofluorescence, Flow cytometry; rabbit anti-phospho-p65 (3033), rabbit anti-phospho-p38 (9215), rabbit anti-phospho-Erk1/2 (9101) are used for mouse, rat monkey and human species for Western blot, Immunoprecipitation, Immunofluorescence, Flow cytometry; rabbit anti-phospho-JNK (4668) is used for mouse, rat and human species for Western blot, Immunoprecipitation, Immunofluorescence, Flow cytometry; rabbit anti-GFP (2956) is used for detection of GFP in Western blot,

Immunoprecipitation, Immunofluorescence, Flow cytometry; rabbit anti-K27 (ab181537), rabbit anti-K48 (8081),rabbit anti-K63 (5621) are used for detection of K27, K48 or K63 Ub in Western blot, Immunoprecipitation, Immunofluorescence, Flow cytometry; rabbit anti-cIAP1 (ab2399) is used for mouse and human species for Western blot; rabbit anti-RNF114 (ab97303, Abcam) is used for mouse and human species for Western blot, Immunofluorescence; Alexa Fluor Plus 488 conjugated goat anti-mouse IgG (A32723), Alexa Fluor Plus 555 conjugated goat anti-mouse IgG (A32727), Alexa Fluor Plus 647 conjugated goat anti-rabbit IgG (A32733) were used for detection of mouse and rabbit IgG in Immunofluorescence; rabbit anti-GST (CW0085M), rabbit anti-His (CW0083M) are used for detection of GST or His for Western blot; rabbit anti-GAPDH (G9545) is used for human, rat and mouse species for Western blot; mouse anti-Flag M2 Affinity Gel (A2220), mouse anti-HA Magnetic Beads (88836), mouse anti-TRAF3 agarose beads (sc-6933 AC) are used for mouse for Immunoprecipitation; goat anti-rabbit IgG (5127), goat anti-mouse IgG (96714) are used for detection of rabbit or mouse IgG in Western blot. rat anti-mouse Ly-6G PE (551461), rat anti-mouse CD11b-FITC (557396) are used for mouse in Flow cytometry.

Efb was detected in Newman (wild-type S. aureus) with western blot using anti-Efb antibody, while none was detected in ΔEfb (knockout Efb in S. aureus) (Supplymentary Fig. 2b).

Eukaryotic cell lines

Policy information about cell lines

Cell line source(s)	HEK293T cells (ATCC CRL-3216) and MH-S cells (ATCC CRL-2019) were obtained from the American Type Culture Collection (ATCC).
Authentication	Cell lines purchased from commercial vendors have been authenticated by the commercial vendor using short tandem repeat (STR)analysis. Gene knockout and knockdown cells were validated by specific antibody.
Mycoplasma contamination	All cell lines were routinely tested for mycoplasma contamination. All cell lines used are free of mycoplasma but not described in text.
Commonly misidentified lines (See ICLAC register)	No commonly misidentified cell lines were used in this study.

Animals and other organisms

Policy information about studies involving animals; ARRIVE guidelines recommended for reporting animal research

Laboratory animals	6 weeks old female SPF C57BL/6 mice were purchased from Beijing HFK Bioscience CO., LTD. Traf3[flox/flox] and Traf3[flox/flox, Lyz2-Cre] mice were purchased from Cyagen Biosciences. All mice were bred under specific pathogen-free (SPF) conditions at the National Engineering Research Center of Immunological Products Animal Center of Army Medical University. All animal experiments were reviewed and approved by the Animal Experiment Administration Committee of Army Medical University and were conducted in accordance with governmental guidelines and institutional policies for the Care and Use of Laboratory Animals. The detail has been described in Methods section (Mice and infection part).
Wild animals	The study did not involve wild animals.
Field-collected samples	The study did not involve samples collected from the field.
Ethics oversight	All animal experiments were reviewed and approved by the Animal Experiment Administration Committee of Army Medical University and were conducted in accordance with governmental guidelines and institutional policies for the Care and Use of Laboratory Animals.

Note that full information on the approval of the study protocol must also be provided in the manuscript.

Flow Cytometry

Plots

Confirm that:

☒ The axis labels state the marker and fluorochrome used (e.g. CD4-FITC).

☒ The axis scales are clearly visible. Include numbers along axes only for bottom left plot of group (a 'group' is an analysis of identical markers).

☒ All plots are contour plots with outliers or pseudocolor plots.

☒ A numerical value for number of cells or percentage (with statistics) is provided.

Methodology

Sample preparation	Neutrophils were obtained from mice blood using anti-Ly6G MicroBeads. Cell surface markers were stained with specific or isotype control antibodies.
Instrument	BD Biosciences FACSCanto
Software	BD Biosciences FACSDiva software

Cell population abundance | The percent of CD11B+LY6G+ cells is 97.2%

Gating strategy | 1. FSC/SSC gate: main cell group; 2. A H: single cell; 3, Fixable viability stain 700: live cells; 4. CD11B+LY6G+: neutrophils.

☒ Tick this box to confirm that a figure exemplifying the gating strategy is provided in the Supplementary Information.
